# Supplementary material for: Global phenotypic profiling identifies a conserved actinobacterial cofactor for a bifunctional PBP-type cell wall synthase
Source: eLife. 2020 Mar 13;9:e54761. doi: 10.7554/eLife.54761 (PMC7205459; doi:10.7554/eLife.54761)
Supplement: Supplementary file 1. — The different growth conditions used for the profiling analysis are listed. Samples 1 g_A, B, and C, correspond to those sequenced to analyze the transposon insertion profile in the original library following one generation of growth. Similarly, 11 g_A and 11 g_B correspond to samples grown for 11 generations without treatment. [file elife-54761-supp1.docx]

| **Supplemental File 1: Growth conditions for the phenotypic profiling.** | | | |  |
| --- | --- | --- | --- | --- |
|  | **Sample name** | **Concentration (µg/mL unless otherwise noted)** | **MIC^C^ (µg/mL unless otherwise noted)** | **Time to read OD 0.5 (hours)** |
|  | 1g_A^a^ |  |  |  |
|  | 1g_B^a^ |  |  |  |
|  | 1g_C^a^ |  |  |  |
|  | 11g_A^b^ |  |  |  |
|  | 11g_B^b^ |  |  |  |
|  | Ampicillin (Amp) | 0.15 | 0.625 | 13.44 |
|  | Ampicillin_H (Amp_H) | 0.2 | 0.625 | 16 |
|  | Bacitracin (Bacit) | 0.1 | 0.125 | 13 |
|  | Cefsulodin (Cef) | 2.5 | 6.25 | 14 |
|  | Cefsulodin_H (Cef_H) | 3 | 6.25 | 15.33 |
|  | Cephalexin (Ceph) | 0.2 | 0.5 | 13 |
|  | Cycloserine (Cyclo) | 37 | 50 | 14 |
|  | Fosfomycin (Fosfo) | 26 | 63 | 12.75 |
|  | Lysozyme (Lyso) | 30 | 125 | 13 |
|  | Lysozyme_H (Lyso_H) | 40 | 125 | 14.75 |
|  | Meropenem (Mero) | 0.04 | 0.078 | 13 |
|  | Meropenem_H (Mero_H) | 0.05 | 0.078 | 16.5 |
|  | Nisin (Nisin) | 1.8 | 10 | 14.5 |
|  | Nisin_H (Nisin_H) | 2 | 10 | 15 |
|  | Penicillin G (PenG) | 0.15 | 0.25 | 14.5 |
|  | Vancomycin (Vanco) | 0.2 | 0.31 | 12 |
|  | Vancomycin_H (Vanco_H) | 0.4 | 0.31 | 14.25 |
|  | Ethambutol (Emb) | 0.2 | 0.5 | 15.66 |
|  | BTZ043 (BTZ043) | 0.045 | 0.08 | 14.3 |
|  | BTZ043_H (BTZ043_H) | 0.05 | 0.08 | 15.5 |
|  | Novobiocin (Novo) | 5 | 12.5 | 13 |
|  | Novobiocin_H (Novo) | 5.5 | 12.5 | 15.15 |
|  | Rifampicin (Rif) | 0.00125 | 0.01 | 12.25 |
|  | Rifampicin_H (Rif_H) | 0.003 | 0.01 | 15 |
|  | Chloramphenicol (Cam) | 0.7 | 1.56 | 15 |
|  | Chloramphenicol_H (Cam_H) | 1 | 1.56 | 15.5 |
|  | Erythromycin (Erythro) | 0.06 | 0.25 | 14.25 |
|  | Erythromycin_H (Erythro_H) | 0.08 | 0.25 | 15.33 |
|  | Streptomycin (Strep) | 0.5 | 0.781 | 14.66 |
|  | Strepomycin_H (Strep_H) | 0.6 | 0.781 | 15.25 |
|  | Tetracycline (Tet) | 0.006 | 0.0125 | 13.5 |
|  | Tetcycline_H (Tet_H) | 0.008 | 0.0125 | 14.5 |
|  | Heat (Heat) | 36° C |  | 11.5 |
|  | MgCl2 (MgCl2) | 150mM | 250mM | 12 |
|  | NaCl (NaCl) | 350mM | 625mM | 12 |
|  | NaCl_H (NaCl_H) | 450mM | 625mM | 14.5 |
|  | SDS (SDS) | 0.0025% | 0.125% | 12.5 |
|  | SDS_H (SDS_H) | 0.0030% | 0.125% | 14.8 |
| ^a^Samples 1g_A, B, and C correspond to those sequenced to analyze the transposon insertion profile in the original library following one generation of growth. | | | | |
| ^b^Samples 11g_A and 11g_B correspond to samples grown for 11 generations without treatment. | | | | |
| ^c^MIC defined as the minimum concentration of drug in a two-fold dilution series required to prevent growth of WT MB001 in a plate reader experiment where cells were grown at 30 degrees in BHI+drug from a starting OD_600_ of 2.5x10^-6^. | | | | |
