# Supplementary material for: Global phenotypic profiling identifies a conserved actinobacterial cofactor for a bifunctional PBP-type cell wall synthase
Source: eLife. 2020 Mar 13;9:e54761. doi: 10.7554/eLife.54761 (PMC7205459; doi:10.7554/eLife.54761)
Supplement: Supplementary file 2. [file elife-54761-supp2.docx]

**Supplemental File 2: Plasmid construction methods**

**pJWS1:** Two DNA fragments corresponding to sequences upstream and downstream of *cgp_3012-cgp_3020* were amplified using two primer pairs: 1)
ATAAATCCTGGTGTCCCTGTTGGATCGAGGTGCATGACCAGGTTG &
GCCCTAATCAGATACGTGACCACATCAGCTACTCCGCACGTG and 2)
ACGTGCGGAGTAGCTGATGTGGTCACGTATCTGATTAGGGC &
CAAGCTTGCATGCCTGCAGGTCGACTCAACTGCGTACATTGCCTTAGC, and MB001 gDNA as the template. The vector was amplified using AGTCGACCTGCAGGCATGCAAGCTTGGCAC &
ATCCAACAGGGACACCAGGATTTATTTATTC as the primers and pCRD206 as the template. The fragments were ligated with the vector by Gibson assembly.

**pJWS2:** Two DNA fragments corresponding to sequences upstream and downstream of *cgp_3019* were amplified using two primer pairs: 1)
ATAAATCCTGGTGTCCCTGTTGGATATGGATTAGGACGAGAGCGGTG &
CGCAAGTTACAGGTTATCGCGCAGAGTTCAACGTGTTCACCATGA and 2)
GTCATGGTGAACACGTTGAACTCTGCGCGATAACCTGTAACTTGC &
CAAGCTTGCATGCCTGCAGGTCGACTCACTGCGTTGCCTTCAAGACC, and MB001 gDNA as the template. The vector was amplified using AGTCGACCTGCAGGCATGCAAGCTTGGCAC &
ATCCAACAGGGACACCAGGATTTATTTATTC as the primers and pCRD206 as the template. The fragments were ligated with the vector by Gibson assembly.

**pJWS3:** Two DNA fragments corresponding to sequences upstream and downstream of *cgp_3018* were amplified using two primer pairs: 1)
ATAAATCCTGGTGTCCCTGTTGGATCTCCACTTTCGCATTGGCATTC &
CATTTAGAACTCACCCTCACCAGGGCCGACAACGAGAACTTTCTTCAT and 2)
ATGAAGAAAGTTCTCGTTGTCGGCCCTGGTGAGGGTGAGTTCTAAATG &
CAAGCTTGCATGCCTGCAGGTCGACTGTGCGCTCCTTACCTGGAATG, and MB001 gDNA as the template. The vector was amplified using AGTCGACCTGCAGGCATGCAAGCTTGGCAC &
ATCCAACAGGGACACCAGGATTTATTTATTC as the primers and pCRD206 as the template. The fragments were ligated with the vector by Gibson assembly.

**pJWS4:** Two DNA fragments corresponding to sequences upstream and downstream of *cgp_0016* were amplified using two primer pairs: 1)
ATAAATCCTGGTGTCCCTGTTGGATCAAATCGCCCAGGTCATCCAG &
GGGGTTTGTGTTTCAGAGGTTAGTCGGATACTTCTCGGGATGCCAT and 2)
ATGGCATCCCGAGAAGTATCCGACTAACCTCTGAAACACAAACCCC &
CAAGCTTGCATGCCTGCAGGTCGACTCCCGATGGAAAGAGACCACC, and MB001 gDNA as the template. The vector was amplified using AGTCGACCTGCAGGCATGCAAGCTTGGCAC &
ATCCAACAGGGACACCAGGATTTATTTATTC as the primers and pCRD206 as the template. The fragments were ligated with the vector by Gibson assembly.

**pJWS18:** NotI-digested pSEC1 was ligated with the insert containing *cgp_0016*, which was amplified from MB001 gDNA using GAAGCTATTACCGCCGCGGCCATGGCATCCCGAGAAGTATCCA &
CGGCCAGTGAATTCACGTGCTTAGTCTACTTCTTCTTGCAGTTGAATC as the primers, by Gibson assembly.

**pJWS19:** NotI-digested pSEC1 was ligated with the insert *cgp_0336 (ponA)*, which was amplified from MB001 gDNA using GAAGCTATTACCGCCGCGGCCGTGTCCACCACGAATTCTCTGA &
CGGCCAGTGAATTCACGTGCCTAGCGGAAGAACTGGTTGATGG as the primers, by Gibson assembly.

**pJWS29:** The insert encoding *Cglu* *ponA* was amplified using MB001 gDNA as the template and the following primers: GGTTCTGGCGGTGGATCCGTGTCCACCACGAATTCTCTGAC &
GATAAGCTTAGTCGACCTCGAGCTAGCGGAAGAACTGGTTGATGGC, and ligated with backbone pHCL149 amplified with CTCGAGGTCGACTAAGCTTATCACC &
GGATCCACCGCCAGAACCTTTA, by Gibson assembly.

**pJWS41:** The insert encoding *linker-cgp_0016* was amplified using pJWS18 as the template and the following primers: CCGGAGGTATGGATGAACTGTATAAGGCTAGCGGTGCTTCGACCC &
CGGATATTATCGTGAGATCGATAAGCTTAGTCTACTTCTTCTTGCAGTTGAATCTGC, and ligated with backbone pHCL152 amplified with GCTTATCGATCTCACGATAATATCCGGG &
CTTATACAGTTCATCCATACCTCCGGTTG, by Gibson assembly.

**pJWS70:** The insert encoding *cofA* from *C. jeikeium* (*jk0012*) was ordered as a gblock from IDT and ligated with backbone pJWS42 amplified with GCTTATCGATCTCACGATAATATCCGGG & GGCCGCGGCGGTAATAGC, by Gibson assembly.

**pJWS73:** The insert encoding *Cglu ponA* transmembrane domain only was amplified using MB001 gDNA as the template and the following primers: GGTTCTGGCGGTGGATCCGTGTCCACCACGAATTCTCTGAC &
CGGTGATAAGCTTAGTCGACCTCGAGCTAACGCGCAACCGCAACGCC, and ligated with backbone pHCL149 amplified with CTCGAGGTCGACTAAGCTTATCACC & GGATCCACCGCCAGAACCTTTA, by Gibson assembly.

**pJWS75:** The insert encoding *Cglu* *ponA* without the transmembrane domain was amplified using MB001 gDNA as the template and the following primers: GGTGCAGGAGAAATTCTCCGGCACGGATGGTCGCGGGC &
GATAAGCTTAGTCGACCTCGAGCTAGCGGAAGAACTGGTTGATGGC, and ligated with backbone pHCL149 amplified with CTCGAGGTCGACTAAGCTTATCACC & GCCGGAGAATTTCTCCTGCACC, by Gibson assembly.

**pJWS78:** The insert encoding *C. jeikeium* *ponA* (*jk1977*) transmembrane domain was ordered as a gblock from IDT and ligated with backbone pHCL149 amplified with CTCGAGGTCGACTAAGCTTATCACC & GCCGGAGAATTTCTCCTGCACC, by Gibson assembly.

**pJWS80:** The insert encoding *Cglu ponB* was amplified using MB001 gDNA as the template and the following primers: GGTTCTGGCGGTGGATCCTTGACGAATAGTAAAAATCCTCCTGCC &
GGTGATAAGCTTAGTCGACCTCGAGTTAGCCGATCCCTAAGAGATCTCCC, and ligated with backbone pHCL149 amplified with CTCGAGGTCGACTAAGCTTATCACC & GGATCCACCGCCAGAACCTTTA, by Gibson assembly.

**pJWS81:** The insert encoding *M. tuberculosis ponA (ponA2)* transmembrane domain was ordered as a gblock from IDT and ligated with backbone pHCL149 amplified with CTCGAGGTCGACTAAGCTTATCACC & GCCGGAGAATTTCTCCTGCACC, by Gibson assembly.

**pJWS83:** The insert encoding M. tuberculosis *cofA* (*rv0007*) was ordered as a gblock from IDT and ligated with backbone pJWS41 amplified with GCTTATCGATCTCACGATAATATCCGGG & GGCCGCGGCGGTAATAGC, by Gibson assembly.

**pJWS88:** The insert encoding *M. tuberculosis ponB (ponA1)* transmembrane domain was ordered as a gblock from IDT and ligated with backbone pHCL149 amplified with CTCGAGGTCGACTAAGCTTATCACC & GCCGGAGAATTTCTCCTGCACC, by Gibson assembly.

**pJWS90:** The insert encoding *C. jeikeium ponB (jk2069)* transmembrane domain was ordered as a gblock from IDT and ligated with backbone pHCL149 amplified with CTCGAGGTCGACTAAGCTTATCACC & GCCGGAGAATTTCTCCTGCACC, by Gibson assembly.

**pJWS97:** The transmembrane domain of ponA was removed from the plasmid pJWS119 by PCR using GGCCGCGGCGGTAATAGCTTCC and ATGCAGACCAACCTTTCAGATCTGACGG. The resulting linear DNA ligated with codon optimized *E. coli* *ponA* transmembrane domain ordered as a gblock from IDT using Gibson assembly.

**pJWS102:** NotI-digested pSEC1 was ligated 2 pieces of *ponA* with the E97A mutation amplified using the primers H443+H956 and H955+H444, by Gibson assembly.

**pJWS103:** The S393A mutation was introduced into *ponA* by inverse PCR pJWS19 using the primers GGTAATGGTGCCGGTGCGATTTTCAAGATCTTT and AAAGATCTTGAAAATCGCACCGGCACCATTACC. The resulting linear DNA was circularized using Gibson assembly.

**pJWS104:** pJWS102 had the S393 mutation introduced into ponA using Agilent QuikChange kit using the primers GGTAATGGTGCCGGTGCGATTTTCAAGATCTTT and AAAGATCTTGAAAATCGCACCGGCACCATTACC.

**pJWS114:** The N terminus of *rv0007* was deleted from pJWS83 by inverse PCR using GAAGCTATTACCGCCGCGGCCATGCAAATCCGCCGCATCG and GGCCGCGGCGGTAATAGC. The resulting linear DNA was circularized using Gibson assembly.

**pJWS119:** The *C. glutamicum* *ponA* with *E. coli* TM was amplified from pJWS97 using primers gttctggcggtggatccGTGAAGTTCGTAAAGTATTTTTTGATCC and gatAAGCTTagtcgacctcgagctaGCGGAAGAACTGGTTGATGGC and was ligated with backbone pHCL149 amplified with ctcgaggtcgactAAGCTTatcacc & ggatccaccgccagaaccttta, by Gibson assembly.
